# Complete Closed Genome Sequences of *Salmonella enterica* subsp. *enterica* Serotypes Anatum, Montevideo, Typhimurium, and Newport, Isolated from Beef, Cattle, and Humans

**DOI:** 10.1128/genomeA.01683-15

**Published:** 2016-02-04

**Authors:** Dayna M. Harhay, James L. Bono, Timothy P. L. Smith, Patricia I. Fields, Blake A. Dinsmore, Monica Santovenia, Christy M. Kelley, Rong Wang, Gregory P. Harhay

**Affiliations:** a USDA ARS U.S. Meat Animal Research Center, Clay Center, Nebraska, USA; b Enteric Diseases Laboratory Branch, Division of Foodborne, Waterborne and Environmental Diseases, Centers for Disease Control and Prevention, Atlanta, Georgia, USA

## Abstract

*Salmonella enterica* spp. are a diverse group of bacteria with a wide range of virulence potential. To facilitate genome comparisons across this virulence spectrum, we present eight complete closed genome sequences of four *S. enterica* serotypes (Anatum, Montevideo, Typhimurium, and Newport), isolated from various cattle samples and from humans.

## GENOME ANNOUNCEMENT

*Salmonella enterica* spp. are a diverse group of bacteria capable of occupying numerous environmental niches and hosts with varying degrees of impact, from commensal colonization to invasive infection. All *S. enterica* are regarded as human pathogens because of their ability to invade intestinal epithelial cells and potential for causing systemic illness (1, 2). However, of the ~1,700 serovars noted for causing human salmonellosis, only 20 account for over 70% of illnesses (3). This may be due to increased risk of exposure to commonly circulating serotypes. However, whole-genome sequence comparisons suggest that some *Salmonella* may possess a greater repertoire of virulence attributes, enabling them to more readily cause human illness (4). To facilitate genome comparisons between *Salmonella* of possibly varying degrees of disease potential, we announce here the availability of complete closed genome sequences of eight *S. enterica* strains, two each of serotypes Anatum, Montevideo, Newport, and Typhimurium, isolated from ground beef, asymptomatic cattle on farm, or at harvest, or from incidences of human salmonellosis.

Genomic DNA was isolated using Qiagen Genomic-tip 100/G columns and a DNA isolation kit (Qiagen, Valencia CA, USA) as per the manufacturer’s protocol. Sequencing was performed on a Pacific Bioscience (PacBio) RS II instrument (Pacific Biosciences, Menlo Park, CA, USA) using P4-C2 or P5-C3 chemistry. Error-corrected sequence reads (>7 kb in length with 14- to 28-fold coverage per genome) were assembled using Celera assembler version 7.0 (5), which produced a single large contig for each isolate, which was then validated and improved using Quiver (6). For all isolates, a self/self dot plot of the consensus sequences revealed at least 3.9-kb overlap between the ends of the contig at >99% identity, which is consistent with a circular chromosome. The duplicated sequence was removed from the 3′-end of each isolate to generate the proper circularized sequence. The origin of replication was approximated using OriFinder (7) and a new, linear model of the chromosome generated using this origin position as base 1. A local instance of Do-It-Yourself Annotator (DIYA) (8) was used to annotate each circularized chromosome.

### Nucleotide sequence accession numbers.

The *S. enterica* genome sequences have been deposited in the NCBI database, and the accession numbers (genome sizes in bp) for strains USDA-ARS-USMARC 1175, 1735, 1899, 1903, and 1927, and CDC strains B94-007410, 2010K-2159, and 2011K-0870 follow, respectively: CP007483 (4,731,586), CP007584 (4,844,415), CP007235 (4,856,450), CP007222 (4,565,462), CP007216 (4,695,590), CP007540 (4,488,371), CP007559 (4,790,387), and CP007523 (4,769,471).
